# On Rest and Pain

**Published:** 1878-12

**Authors:** 


### BIBLIOGRAPHICAL.

---

ON REST AND PAIN. By John Hilton, F.R.S., F.R.C.S. Second edition. New York: Wm. Wood & Co., 1879. pp. 299.

Messrs. Wm. Wood & Co., sometime ago, announced their intention of publishing a series of standard medical works in a cheap form to appear in monthly volumes, and to be known as Wood's Library of Standard Medical Authors. The above work is the first of the series, and will be followed by others of similar practical value.

